# Real-world outcomes following biochemical recurrence after definitive therapy with a short prostate-specific antigen doubling time: potential role of early secondary treatment

**DOI:** 10.1038/s41391-024-00894-0

**Published:** 2024-09-13

**Authors:** Stephen J. Freedland, Wei Gao, Angela Lax, Hongbo Yang, Krishnan Ramaswamy, David Russell, Agnes Hong, Jasmina I. Ivanova

**Affiliations:** 1 Cedars-Sinai Cancer Center, Los Angeles, CA USA; 2 The Durham VA Medical Center, Durham, NC USA; 3 Analysis Group Inc., Boston, MA USA; 4 Pfizer Inc., New York, NY USA

**Keywords:** Prostate cancer, Cancer therapy, Prostate cancer, Outcomes research, Prostate cancer

## Abstract

**Background:**

The natural history of biochemical recurrence (BCR) managed with delayed hormonal therapy is well documented by data from Johns Hopkins. However, as many patients receive treatment prior to metastasis, we evaluated the natural history and role of prostate-specific antigen doubling time (PSADT) in a more contemporary cohort of BCR patients with nonmetastatic castration-sensitive prostate cancer (nmCSPC).

**Methods:**

Patients in the Veterans Health Administration (VHA; 01/01/06 to 06/22/20) with nmCSPC and BCR were divided into rapid ( ≤9 months) and less rapid ( >9 to ≤15 months) PSADT cohorts. Patients with PSADT >15 months were excluded as outcomes, even with delayed treatment, are excellent. Outcomes included time to first antineoplastic therapy after BCR, metastasis, metastasis-free survival (MFS), and overall survival (OS). Cox models adjusted for baseline demographics and clinical characteristics.

**Results:**

Overall, 781 patients with BCR were identified (502 rapid; 279 less rapid PSADT). Rapid PSADT was associated with shorter time to first systemic antineoplastic therapy (median 11.4 vs. 28.3 months, adjusted hazard ratio [95% confidence interval] 2.17 [1.83–2.57]), metastasis (102.4 months vs. not reached, 1.79 [1.33–2.40]), MFS (76.1 vs. 106.3 months, 1.73 [1.33–2.24]), and OS (120.5 vs. 140.5 months, 1.76 [1.22–2.54]) versus less rapid PSADT.

**Conclusion:**

Most patients with rapid PSADT underwent secondary treatment within 1 year after BCR. More contemporary patients treated with early secondary treatment had better outcomes than historical data from patients who had delayed treatment. Whether these results reflect the benefits of early secondary treatment or overall improvements in prostate cancer outcomes over time requires further study.

## Introduction

Prostate cancer progresses through a series of characteristic clinical states reflecting the natural history of the disease and response to treatment. Following initial diagnosis and treatment, patients with prostate cancer may experience rising prostate-specific antigen (PSA) levels or biochemical recurrence (BCR). Despite BCR, most patients do not develop metastases or die from prostate cancer after primary therapy [1, 2], but are at an increased risk of prostate cancer-related morbidity and mortality [3].

Real-world data on treatment patterns and clinical outcomes are limited for patients with nonmetastatic castration-sensitive prostate cancer (nmCSPC) and BCR after primary therapy. Current understanding of the natural history of BCR largely comes from treatment data from the Johns Hopkins Hospital [3, 4]. These studies included patients with BCR following radical prostatectomy (RP) and most patients received delayed androgen deprivation therapy (ADT) at metastasis, with few patients receiving early ADT (prior to metastasis). Among all BCR patients, regardless of PSA doubling time (PSADT), median time from BCR to metastasis was 8 years. However, this was dependent on PSADT, with worse outcomes reported for shorter PSADT (especially <3 months) [4]. These data contributed to guidelines recommending observation for patients with low-risk disease with nmCSPC and BCR after primary therapy, with salvage treatment or intermittent ADT recommended for high-risk disease [5–8]. However, real-world data show that most patients who receive ADT after BCR do so prior to metastases [9]. The benefits of early ADT are unclear. In TOAD, early ADT improved overall survival (OS) in patients with BCR or non-curable prostate cancer compared with delayed ADT [10]. However, when results were combined with ELAAT, no OS differences were observed [11]. Nonetheless, the natural history of early treatment with ADT, and whether PSADT remains as equally prognostic in this population, is unclear. This is especially relevant for patients with a shorter PSADT of <15 months as when managed with delayed ADT, they have a higher risk of metastasis and death related to prostate cancer than patients with longer PSADT. In contrast, outcomes among patients with a PSADT of >15 months are excellent, even without secondary treatments [1]. Hence, we limited our study to high-risk BCR to better understand the timing of secondary treatments and the impact of PSADT on long-term outcomes in more contemporary patients who often received early ADT.

This study of Veteran’s Health Administration (VHA) data aimed to assess real-world outcomes in patients with nmCSPC as a function of PSADT following BCR after definitive treatment and to help put our findings within the context of prior studies, using historical controls of patients largely treated with delayed ADT.

## Methods

### Data source

This retrospective, observational study of patients with nmCSPC and BCR after definitive therapy utilized data from the VHA database (January 1, 2006 to June 22, 2020). The VHA is the largest integrated healthcare system in the United States, providing care to over 9 million veterans [12]. The study design was determined to be exempt from institutional review board review by the Southeast Louisiana Veterans Health Care System institutional review board.

### Study cohort

Included patients were aged ≥18 years, with ≥1 diagnosis of prostate cancer, had previously received definitive treatment with RP or external radiotherapy after diagnosis, with evidence of BCR, and no evidence of metastasis any time before or within 90 days after the index date. The index date was the date of BCR, defined as the date of the first PSA value that met the following: patients who underwent RP had to have ≥1 PSA value ≥1 ng/mL >30 days after RP; patients who received external radiotherapy only, without RP, were required to have one PSA value ≥2 ng/mL above the nadir, defined as the lowest PSA value from the end of the first observed radiotherapy to this PSA measurement. Patients were required to have a PSADT of ≤15 months. PSADT was calculated as the natural logarithm of two divided by the slope obtained from fitting a linear regression of the natural log of PSA over time. Further details on the inclusion criteria can be found in Supplementary Fig. S1. Patients were divided into two cohorts: patients with rapid PSADT ( ≤9 months) and less rapid PSADT (>9 to ≤15 months). A PSA cutoff of ≥1 ng/mL for BCR and ≤9 months for rapid PSADT were selected as these were the cutoffs used for EMBARK to identify patients with BCR after RP and that had high-risk disease, respectively, and are endorsed by the U.S. Food and Drug Administration to define high-risk BCR [13].

### Patient characteristics and study outcomes

Patient demographics and clinical characteristics were measured during the baseline period (1 year prior to index). Treatment patterns and clinical outcomes were investigated during the follow-up period (from index to the end of continuous eligibility or death, whichever occurred first). Treatment patterns were described as the time to first antineoplastic therapy after BCR. The antineoplastic therapies considered included ADT, novel hormonal therapy (NHT), first-generation nonsteroidal antiandrogen (NSAA), chemotherapy, sipuleucel-T, pembrolizumab, radium 223, olaparib, rucaparib, and salvage radiotherapy. The clinical outcomes assessed included time to metastasis (time from index date to the first diagnosis of metastatic disease), metastasis-free survival (MFS; time from index to the first diagnosis of metastatic disease or death), and OS (time from index to death). If patients did not have evidence of metastasis, they were censored at the end of enrollment, end of data, or death, whichever was earliest. Metastasis was defined using International Classification of Diseases (ICD)-9-CM codes 196–199.1 or ICD-10 codes C77, C78, C79, and C7B. Patients with no evidence of metastasis or death were censored at end of enrollment or data end, whichever was earlier. If patients did not die during the follow-up period, they were censored at the end of enrollment or data end, whichever was earliest.

### Statistical analysis

Descriptive analyses were conducted for baseline demographics and clinical characteristics by cohort. Unadjusted comparisons of baseline characteristics between rapid and less rapid PSADT cohorts were conducted using standardized mean difference. Kaplan–Meier analysis was used to describe the time from index to first antineoplastic therapy after BCR and time to clinical event outcomes. Outcomes were compared between PSADT cohorts using unadjusted and adjusted Cox proportional hazards models accounting for age, age group (<60 [reference], 60–69, 70–79, and ≥80 years), race (White [reference], Black, Hispanic, and other), log(time from definitive therapy to index date), log(index PSA), modified Charlson Comorbidity Index, and index year (2006–2016 vs. 2017–2020). Exploratory analyses for MFS and OS by further PSADT cohorts (≤3, >3 to ≤9, >9 to ≤12, and >12 to ≤15 months) were conducted. Analyses were conducted using SAS version 9.4 (SAS Institute, Cary, NC, USA).

## Results

### Baseline demographics and clinical characteristics

In total, 781 patients with nmCSPC and BCR were included (Supplementary Fig. S1); 502 had rapid PSADT and 279 had less rapid PSADT. Patients with rapid PSADT were more likely to be younger, White, and have a shorter mean time from definitive therapy to index than patients with less rapid PSADT (Table 1).

**Table 1:** Baseline patient demographics and clinical characteristics.

	Overall (*N* = 781)	Less rapid PSADT cohort (i.e., PSADT >9 months and ≤15 months) (*n* = 279)	Rapid PSADT cohort (i.e., PSADT ≤9 months) (*n* = 502)	Standardized difference (%)^†^
**Demographics at index date**^**‡**^				
Age, mean ± SD (median) [IQR], years	66.7 ± 6.6 (67.1) [62.9–70.6]	67.3 ± 6.1 (67.6) [63.7–71.0]	66.4 ± 6.8 (66.7) [62.3–70.5]	–13.79*
Age categories, *n* (%), years				
18–49	7 (0.9)	1 (0.4)	6 (1.2)	9.54
50–59	109 (14.0)	29 (10.4)	80 (15.9)	16.45*
60–69	445 (57.0)	166 (59.5)	279 (55.6)	–7.94
70–79	198 (25.4)	73 (26.2)	125 (24.9)	–2.90
≥80	22 (2.8)	10 (3.6)	12 (2.4)	–7.02
Race,^§^ *n* (%)				
White, non-Hispanic	393 (50.3)	130 (46.6)	263 (52.4)	11.61*
Black	282 (36.1)	106 (38.0)	176 (35.1)	–6.09
Hispanic	52 (6.7)	21 (7.5)	31 (6.2)	–5.35
Native American	10 (1.3)	4 (1.4)	6 (1.2)	–2.09
Asian	1 (0.1)	1 (0.4)	0 (0.0)	–8.48
Other	4 (0.5)	1 (0.4)	3 (0.6)	3.47
Unknown	39 (5.0)	16 (5.7)	23 (4.6)	–5.22
Geographic regions^‖^, *n* (%)				
South	209 (26.8)	78 (28.0)	131 (26.1)	–4.19
Midwest	249 (31.9)	87 (31.2)	162 (32.3)	2.34
West	241 (30.9)	83 (29.7)	158 (31.5)	3.74
Northeast	82 (10.5)	31 (11.1)	51 (10.2)	–3.09
Index year distribution, *n* (%)				
2006	5 (0.6)	1 (0.4)	4 (0.8)	5.79
2007	14 (1.8)	5 (1.8)	9 (1.8)	0.01
2008	20 (2.6)	5 (1.8)	15 (3.0)	7.84
2009	28 (3.6)	10 (3.6)	18 (3.6)	0.01
2010	41 (5.2)	10 (3.6)	31 (6.2)	12.05*
2011	46 (5.9)	15 (5.4)	31 (6.2)	3.43
2012	58 (7.4)	24 (8.6)	34 (6.8)	–6.87
2013	59 (7.6)	23 (8.2)	36 (7.2)	–4.02
2014	74 (9.5)	31 (11.1)	43 (8.6)	–8.55
2015	73 (9.3)	33 (11.8)	40 (8.0)	–12.95*
2016	92 (11.8)	29 (10.4)	63 (12.5)	6.77
2017	93 (11.9)	24 (8.6)	69 (13.7)	16.38*
2018	99 (12.7)	42 (15.1)	57 (11.4)	–10.94*
2019	68 (8.7)	23 (8.2)	45 (9.0)	2.57
2020	11 (1.4)	4 (1.4)	7 (1.4)	–0.33
**Clinical characteristics**				
Time from definitive therapy (i.e., first observed radical prostatectomy or external beam radiotherapy) to index date (months), mean ± SD (median) [IQR]	44.9 ± 35.1 (39.3) [15.3–68.1]	51.8 ± 36.2 (48.5) [21.9–77.2]	41.0 ± 33.9 (32.3) [12.6–62.5]	–30.56*
Medication and procedure history^¶^, *n* (%)				
Radical prostatectomy only				
Any time prior to index date	583 (74.6)	209 (74.9)	374 (74.5)	–0.94
Baseline period	143 (18.3)	45 (16.1)	98 (19.5)	8.87
External beam radiotherapy only				
Any time prior to index date	142 (18.2)	53 (19.0)	89 (17.7)	–3.27
Baseline period	48 (6.1)	11 (3.9)	37 (7.4)	14.88*
Radical prostatectomy and external beam radiotherapy^#^				
Any time prior to index date	56 (7.2)	17 (6.1)	39 (7.8)	6.60
Baseline period	1 (0.1)	0 (0.0)	1 (0.2)	6.32
Pain medication	22 (2.8)	10 (3.6)	12 (2.4)	–7.02
Chronic corticosteroid use^††^	1 (0.1)	1 (0.4)	0 (0.0)	–8.48
Bone protective agents	1 (0.1)	1 (0.4)	0 (0.0)	–8.48
Prognostic variables^‡‡^, mean ± SD (median) [IQR]				
PSA on the index date, *n* (%)	781 (100.0)	279 (100.0)	502 (100.0)	0.00
PSA value, ng/mL	4.4 ± 44.4 (1.5) [1.2–2.6]	2.7 ± 5.6 (1.4) [1.1–2.4]	5.3 ± 55.3 (1.6) [1.2–2.8]	6.60
Hemoglobin (last measurement within 180 days prior to or on the index date), *n* (%)	583 (74.6)	214 (76.7)	369 (73.5)	–7.40
Hemoglobin value, g/dL	13.9 ± 1.8 (14.0) [12.8–15.0]	14.0 ± 1.7 (14.1) [13.0–15.1]	13.8 ± 1.8 (13.9) [12.7–15.0]	–12.90*
Alkaline phosphatase (last measurement within 180 days prior to or on the index date), *n* (%)	511 (65.4)	174 (62.4)	337 (67.1)	9.99
Alkaline phosphatase value, IU/L	79.8 ± 32.6 (75.0) [62.0–91.0]	80.0 ± 27.1 (75.0) [63.0–95.0]	79.7 ± 35.2 (75.0) [62.0–90.0]	–1.21
Modified NCI Charlson Comorbidity Index (excluding cancer), mean ± SD (median) [IQR]	1.3 ± 1.5 (1.3) [0.0–1.7]	1.3 ± 1.6 (1.3) [0.0–1.7]	1.3 ± 1.5 (1.3) [0.0–1.7]	–0.33
Individual comorbidities, *n* (%)				
Hypertension	507 (64.9)	176 (63.1)	331 (65.9)	5.97
Hyperlipidemia	411 (52.6)	150 (53.8)	261 (52.0)	–3.55
Impotence	245 (31.4)	90 (32.3)	155 (30.9)	–2.97
Diabetes	216 (27.7)	81 (29.0)	135 (26.9)	–4.77
Urinary tract infection	116 (14.9)	41 (14.7)	75 (14.9)	0.69
Chronic obstructive pulmonary disease	98 (12.5)	31 (11.1)	67 (13.3)	6.83
Arrhythmia	36 (4.6)	10 (3.6)	26 (5.2)	7.80
Congestive heart failure	37 (4.7)	10 (3.6)	27 (5.4)	8.68
Myocardial infarction	20 (2.6)	8 (2.9)	12 (2.4)	–2.98
Stroke	27 (3.5)	11 (3.9)	16 (3.2)	–4.07
Angina pectoris	10 (1.3)	3 (1.1)	7 (1.4)	2.89
Inflammatory bowel disease	6 (0.8)	1 (0.4)	5 (1.0)	7.78
Acute coronary syndrome	7 (0.9)	3 (1.1)	4 (0.8)	–2.89
Low-extremity arterial occlusive disease	2 (0.3)	1 (0.4)	1 (0.2)	–3.02

^†^The standardized difference was multiplied by 100 to get the percent standardized difference. A value >10% is considered a significant imbalance. ^‡^The baseline period was defined as the 365 days prior to the index date. The index date was defined as the first PSA value to meet the definition of BCR. Mean ± SD (median) follow-up time among all patients was 55.4 ± 34.3 (49.2) months, and was 57.4 ± 54.5 (34.2) months in the less rapid PSADT cohort and 54.2 ± 34.3 (46.3) months in the rapid PSADT cohort. ^§^The race reported during the index year was used. ^‖^The region documented during the index year was used. Midwest includes IA, IL, IN, KS, MI, MN, MO, ND, NE, OH, SD, and WI; Northeast includes CT, MA, ME, NH, NJ, NY, PA, RI, and VT; South includes AL, AR, DC, DE, FL, GA, KY, LA, MD, MS, NC, OK, SC, TN, TX, VA, and WV; West includes AK, AZ, CA, CO, HI, ID, MT, NM, NV, OR, UT, WA, and WY. ^¶^Medication and procedure history is described during the baseline period, unless otherwise specified. ^#^Patients may have received both radical prostatectomy and external beam radiotherapy prior to the index date. ^††^Chronic corticosteroid use was defined as a treatment duration of ≥90 days with a gap of no more than 30 days between consecutive pharmacy claims (i.e., <30 days between the end of the days’ supply of one claim and the start of the next claim). ^‡‡^PSA on the index date was summarized. For hemoglobin and alkaline phosphatase, the measurement within 180 days prior to the index date (including the index date) was summarized. If there were multiple labs within this period, the measurement closest to the index date was summarized. *Standardized difference >10%. *BCR* biochemical recurrence; *IQR* interquartile range; *NCI* National Cancer Institute; *PSA* prostate-specific antigen; *PSADT* PSA doubling time; *SD* standard deviation.

### Treatment patterns

**Fig. 1:**
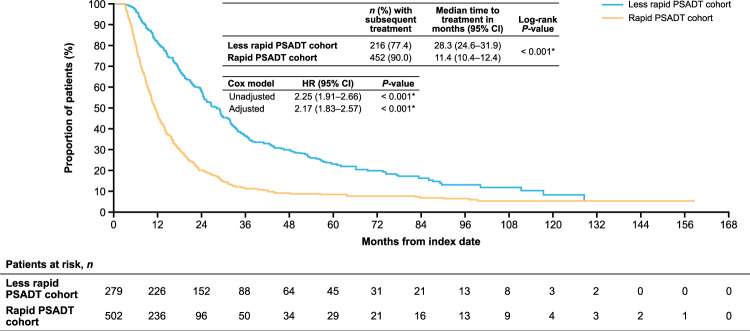
Time to first systemic antineoplastic therapy after BCR among patients with nmCSPC by PSADT cohort. The adjusted Cox proportional hazards models adjusted for patient demographics and clinical characteristics including age, age group (<60 [reference], 60–69, 70–79, and ≥80 years), race (White [reference], Black, Hispanic, and other), log(time from definitive therapy to index date), log(index PSA), modified Charlson Comorbidity Index, and index year (2006–2016 vs. 2017–2020). *P-value ≤ 0.05. *BCR* biochemical recurrence; *CI* confidence interval; *HR* hazard ratio; *nmCSPC* nonmetastatic castration-sensitive prostate cancer; *PSA* prostate-specific antigen; *PSADT* PSA doubling time.

Median follow-up time after BCR was 49.2 months for all patients. During follow-up after BCR, 90% (*n* = 452) of patients with rapid PSADT and 77% (*n* = 216) with less rapid PSADT received systemic antineoplastic therapy. Among treated patients, ADT plus first-generation NSAA and ADT alone were the most common systemic antineoplastic therapies, followed by first-generation NSAA alone for both patients with rapid and less rapid PSADT (Supplementary Table S1). Median time to first systemic antineoplastic therapy was <1 year for patients with rapid PSADT, which was shorter versus patients with less rapid PSADT, where median time was >2 years (11.4 months, 95% confidence interval [CI] 10.4–12.4 vs. 28.3 months, 95% CI 24.6–31.9; *P* < 0.001; adjusted hazard ratio [HR] 2.17, 95% CI 1.83–2.57; *P* < 0.001; Fig. 1).

### Time to metastasis

**Fig. 2:**
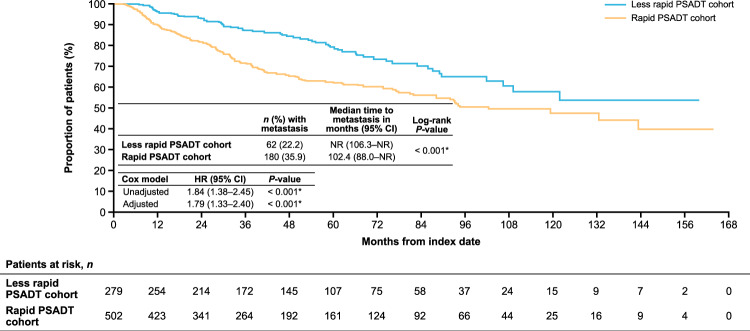
Time to metastasis after BCR among patients with nmCSPC by PSADT cohort. The adjusted Cox proportional hazards models adjusted for patient demographics and clinical characteristics including age, age group (<60 [reference], 60–69, 70–79, and ≥80 years), race (White [reference], Black, Hispanic, and other), log(time from definitive therapy to index date), log(index PSA), modified Charlson Comorbidity Index, and index year (2006–2016 vs. 2017–2020). *P-value ≤ 0.05. *BCR* biochemical recurrence; *CI* confidence interval; *HR* hazard ratio; *nmCSPC* nonmetastatic castration-sensitive prostate cancer; *NR* not reached; *PSA* prostate-specific antigen; *PSADT* PSA doubling time.

Thirty-six percent (*n* = 180) of patients with rapid PSADT and 22% (*n* = 62) with less rapid PSADT developed metastasis during follow-up. The median time to metastasis was shorter for patients with rapid PSADT versus less rapid PSADT (102.4 months, 95% CI 88.0–not reached [NR] vs. NR months, 95% CI 106.3–NR; *P* < 0.001; adjusted HR 1.79, 95% CI 1.33–2.40; *P* < 0.001; Fig. 2).

### Metastasis-free survival

**Fig. 3:**
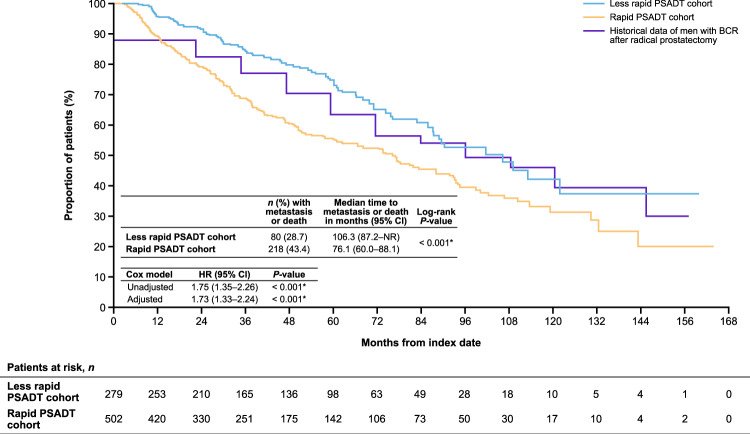
MFS after BCR among patients with nmCSPC by PSADT cohort including historical Johns Hopkins data. Includes historical metastasis-free survival data of patients with BCR after radical prostatectomy from Pound et al. 1999 [3]. The adjusted Cox proportional hazards models adjusted for patient demographics and clinical characteristics including age, age group (<60 [reference], 60–69, 70–79, and ≥80 years), race (White [reference], Black, Hispanic, and other), log(time from definitive therapy to index date), log(index PSA), modified Charlson Comorbidity Index, and index year (2006–2016 vs. 2017–2020). *P-value ≤ 0.05. *BCR* biochemical recurrence; *CI* confidence interval; *HR* hazard ratio; *MFS* metastasis-free survival; *nmCSPC* nonmetastatic castration-sensitive prostate cancer; *PSA* prostate-specific antigen; *PSADT* PSA doubling time.

Forty-three percent (*n* = 218) of patients with rapid PSADT and 29% (*n* = 80) with less rapid PSADT developed metastasis or died during follow-up. The median time to metastasis or death was shorter for patients with rapid PSADT versus less rapid PSADT (76.1 months, 95% CI 60.0–88.1 vs. 106.3 months, 95% CI 87.2–NR; *P* < 0.001; adjusted HR 1.73, 95% CI 1.33–2.24; *P* < 0.001; Fig. 3). Risk of metastasis or death was significantly higher for PSADT ≤3 and >3 to ≤9 months versus >12 to ≤15 months (*P* < 0.001 and *P* = 0.012, respectively; Supplementary Fig. S2).

### Overall survival

**Fig. 4:**
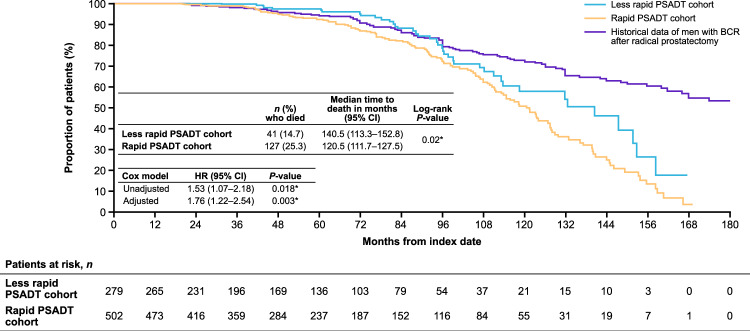
OS after BCR among patients with nmCSPC by PSADT cohort including historical Johns Hopkins data. Includes historical overall survival data of patients with BCR after radical prostatectomy from Freedland et al. 2007 [4]. The adjusted Cox proportional hazards models adjusted for patient demographics and clinical characteristics including age, age group (<60 [reference], 60–69, 70–79, and ≥80 years), race (White [reference], Black, Hispanic, and other), log(time from definitive therapy to index date), log(index PSA), modified Charlson Comorbidity Index, and index year (2006–2016 vs. 2017–2020). *P-value ≤ 0.05. *BCR* biochemical recurrence; *CI* confidence interval; *HR* hazard ratio; *nmCSPC* nonmetastatic castration-sensitive prostate cancer; *OS* overall survival; *PSA* prostate-specific antigen; *PSADT* PSA doubling time.

Twenty-five percent (*n* = 127) of patients with rapid PSADT and 15% (*n* = 41) with less rapid PSADT died during follow-up. The median time to death was shorter for patients with rapid PSADT versus less rapid PSADT (120.5 months, 95% CI 111.7–127.5 vs. 140.5 months, 95% CI 113.3–152.8; *P* = 0.02; adjusted HR 1.76, 95% CI 1.22–2.54; *P* = 0.003; Fig. 4). Risk of death was significantly higher for PSADT ≤3 months versus >12 to ≤15 months (*P* < 0.001; Supplementary Fig. S3).

## Discussion

This study of VHA data over a 15-year period demonstrates that most patients with rapid PSADT underwent secondary treatment within 1 year after BCR, and over 2 years for patients with less rapid PSADT. Intriguingly, outcomes among patients with rapid PSADT compared favorably to prior studies of all BCR patients treated with ADT delayed until metastasis [3]. Further study will determine whether this reflects the benefits of early aggressive treatment of high-risk BCR or improved outcomes over time.

Natural history studies of patients with ADT delayed until metastasis show the important prognostic value of PSADT [3, 4]. Importantly, patients with rapid PSADT had worse time to metastasis, MFS, and OS compared with patients who had less rapid PSADT. Our results mirror these findings but extend the results to those who largely received early aggressive secondary treatment for BCR. Our exploratory analyses found that patients with PSADT ≤3 months and PSADT >3 to ≤9 months had worse MFS and patients with PSADT ≤3 months had worse OS than patients with PSADT >12 to ≤15 months. These results further support the prognostic role of PSADT, regardless of whether patients receive early or delayed ADT.

The median time to metastasis for patients with rapid PSADT was 102 months (8.5 years). Similarly, there were 8 years until metastatic spread in patients with BCR in the Johns Hopkins analyses, none of whom received secondary hormonal therapy prior to metastasis [3]. Importantly, the Johns Hopkins analysis included *all* BCR patients, not just those who were high-risk. For those with PSADT <10 months in the Johns Hopkins data, median time to metastatic disease was around 5 years [3]. As >80% of our cohort underwent prior surgery, it is noteworthy that we defined BCR as a PSA value of ≥1 ng/mL, which potentially excludes patients for whom salvage therapy is a treatment option. This cutoff is higher than that used in prior Johns Hopkins data, where BCR was defined as a PSA value of ≥0.2 ng/mL [3]. As such, despite being further along in their BCR journey, our patients had better outcomes. One possible explanation is that early secondary treatment of high-risk BCR improves outcomes. Alternatively, as new therapies are introduced and stage migration continues, overall outcomes in prostate cancer are improving [14]. Given the retrospective nature of our study, we cannot imply cause and effect, and comparison across studies is challenging. More studies are needed to understand the reasons for better outcomes in more contemporary patients treated with aggressive, early, secondary therapy.

While comparing time to metastatic disease is important, it is possible that many of the metastases that developed in our study were castration-resistant at the time of metastases versus the Johns Hopkins analysis where they were castration-sensitive. Therefore, it is important to also compare OS. The median OS in patients with rapid PSADT in our study (120.5 months [10.0 years]) aligned with previous results in patients with a PSADT of 3 to 8.9 months from longer term follow-up from Johns Hopkins [4]. However, our cohort was older than the Johns Hopkins cohort (median age 66.4 and 61.5 years, respectively; see Supplementary Fig. S4 for general male-specific mortality by age) and the Johns Hopkins PSADT 3 to 8.9 month group did not include patients with PSADT <3 months, unlike our PSADT ≤9 month group which included these very high-risk patients. In contrast, patients with less rapid PSADT in our series had a median OS of 140.5 months (11.7 years), whereas median OS was not reached at 15 years in the Johns Hopkins data for patients with a PSADT of 9 to 14.9 months. Notably, the risk of prostate cancer death in patients with slower PSADT is much lower than those with more rapid PSADT and, therefore, it is likely that many deaths in our study were not related to prostate cancer. As such, the worse outcomes in our patients with less rapid PSADT suggest that our cohort had poorer overall health versus the Johns Hopkins cohort. When taken together with the older age, inclusion of a very high-risk group (PSADT < 3 months), and higher PSA levels at BCR within our cohort, this further emphasizes the potential clinical significance of the similar median OS between patients with rapid PSADT in our study and historical Johns Hopkins data [4]. Again, whether this reflects the benefit of early ADT in patients with high-risk nmCSPC and BCR or temporal changes in the natural history over time requires investigation in prospective randomized trials.

Several studies investigated the clinical benefit of adding chemotherapy or NHT to ADT in patients with high-risk BCR. TAX3503 did not demonstrate a meaningful benefit of adding docetaxel to ADT in patients with high-risk BCR [15]. One possible interpretation of our data is that early ADT is beneficial; if true, it is possible that intensified ADT may provide further benefit. Indeed, EMBARK demonstrated that enzalutamide, with or without ADT, improved MFS and the time to first use of new antineoplastic therapy compared with placebo plus ADT in patients with high-risk nmCSPC and BCR (PSADT ≤9 months) following definitive therapy (RP or radiotherapy) [13]. Additionally, ADT plus apalutamide, with or without abiraterone, prolonged biochemical progression-free survival in patients with high-risk BCR following RP in AFT-19 [16]. Follow-up is ongoing to determine longer-term outcomes, including OS in EMBARK, and MFS and time to castration resistance in AFT-19.

Our findings may not be generalizable to populations other than patients from the VHA and are limited by possible incorrect diagnosis coding. Furthermore, care occurring outside the VHA may not have been captured, which could have led to misclassifications of disease states or treatments. Additionally, a prescription may not signify that the medication was taken as prescribed. As we excluded patients with ADT at any point prior to index to ensure that PSADT calculations were free from any lingering effects of ADT, patients who received standard-of-care ADT plus radiotherapy were not included. As such, RP patients are likely over-represented in our cohort, though this permits closer comparisons to prior Johns Hopkins studies that included only RP patients. Our definition of BCR was more restrictive than the Johns Hopkins studies, which defined BCR as a PSA value of ≥0.2 ng/mL. Finally, our OS estimates also reflect the availability of life-prolonging therapies for metastatic disease that were not available during the historical Johns Hopkins analyses.

In summary, patients with nmCSPC, BCR, and rapid PSADT following definitive therapy in the VHA had shorter time to first systemic antineoplastic therapy and worse outcomes for time to metastasis, MFS, and OS, than patients with less rapid PSADT. Patients with rapid PSADT from the VHA, who were heavily treated with secondary treatment, older, had potentially poorer health, and had higher PSA levels at BCR than patients from the historical Johns Hopkins data, had similar outcomes to patients who did not receive aggressive secondary treatment. This suggests either a potential benefit of aggressive, early treatment of patients with rapid PSADT after BCR and/or temporal improvements in outcomes over time. Further study exploring the relationship between short PSADT and lineage plasticity may be warranted.

## Supplementary information

Supplemental Material

Video EPC

## Data Availability

As the data supporting the findings of this study were used under license for the current study, restrictions apply to the authors’ ability to make data publicly available. The data are available from the Veterans Health Administration.
