# Supplementary material for: Real-world outcomes following biochemical recurrence after definitive therapy with a short prostate-specific antigen doubling time: potential role of early secondary treatment
Source: Prostate Cancer Prostatic Dis. 2024 Sep 13;28(3):809–16. doi: 10.1038/s41391-024-00894-0 (PMC12399425; doi:10.1038/s41391-024-00894-0)

Supplementary Table S1. First systemic antineoplastic therapy after BCR by PSADT cohort.

| **First regimen received after index date^†^** | **Less rapid PSADT cohort (*n* = 279)** | **Rapid PSADT cohort  (*n* = 502)** |
| --- | --- | --- |
| *N* (%) with event | 216 (77.4) | 452 (90.0) |
| ADT + first-generation NSAA | 76 (35.2) | 163 (36.1) |
| ADT only | 74 (34.3) | 164 (36.3) |
| First-generation NSAA only | 54 (25.0) | 112 (24.8) |
| ADT + NHT + first-generation NSAA | 5 (2.3) | 2 (0.4) |
| NHT only | 3 (1.4) | 4 (0.9) |
| ADT + NHT | 2 (0.9) | 5 (1.1) |
| Chemotherapy only | 1 (0.5) | 1 (0.2) |
| ADT + chemotherapy | 1 (0.5) | 0 (0.0) |
| NHT + first-generation NSAA | 0 (0.0) | 1 (0.2) |

^†^Treatments received within 28 days of first treatment after the index date were included in the same regimen.
*ADT* androgen deprivation therapy; *BCR* biochemical recurrence; *NHT* novel hormonal therapy; *NSAA* nonsteroidal anti-androgen; *PSA* prostate-specific antigen; *PSADT* PSA doubling time.

Supplementary Fig. S1. Identification of analysis population.

^
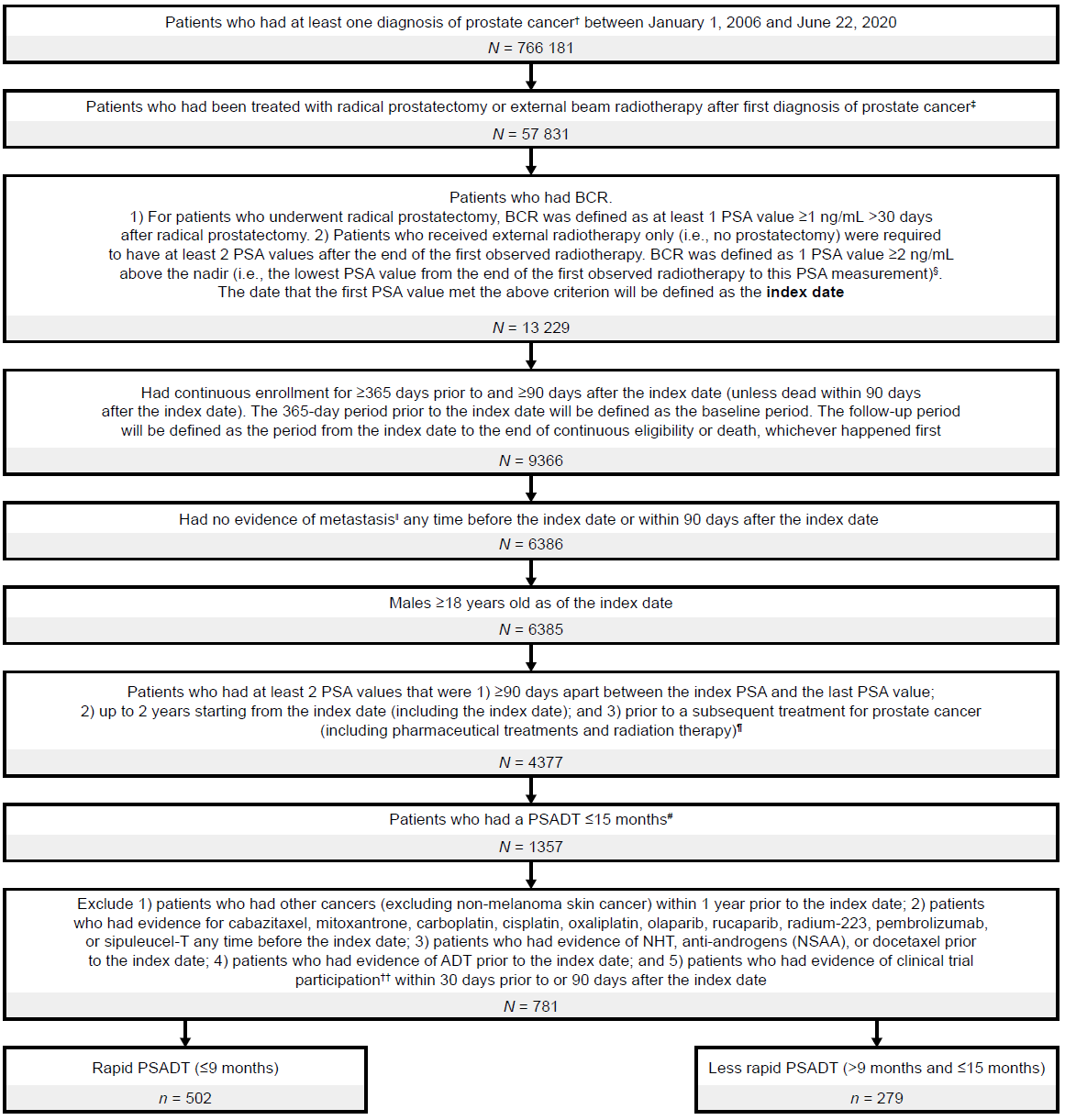
^

^†^Diagnosis of prostate cancer was identified using ICD-9-CM diagnosis code (185) and ICD-10-CM diagnosis code (C61).
^‡^Radical prostatectomy and external radiotherapy were identified using administrative codes.
^§^Discontinuation of radiotherapy was defined as a gap of ≥30 days between two radiotherapy claims.
^‖^Metastases were identified via ICD-9-CM codes 196–199.1 or ICD-10 codes C77, C78, C79, and C7B.
^¶^Treatments for prostate cancer include ADT, NHT, first-generation NSAA, definitive therapy, chemotherapy, immunotherapy, radium, radiation therapy, and poly (adenosine diphosphate-ribose) polymerase inhibitors.
^#^PSADT was calculated as the natural logarithm of two divided by the slope obtained from fitting a linear regression of the natural log of PSA on time. The calculation utilized all PSA values within 2 years starting from the index date and prior to a subsequent treatment for prostate cancer.
^††^Clinical trial participation was identified based on ICD-10 CM code Z00.6 and ICD-9 CM code V70.7.
*ADT* androgen deprivation therapy; *BCR* biochemical recurrence; *ICD* International Classification of Disease; *NHT* novel hormonal therapy; *NSAA* nonsteroidal anti-androgen; *PSA* prostate-specific antigen; *PSADT* PSA doubling time.


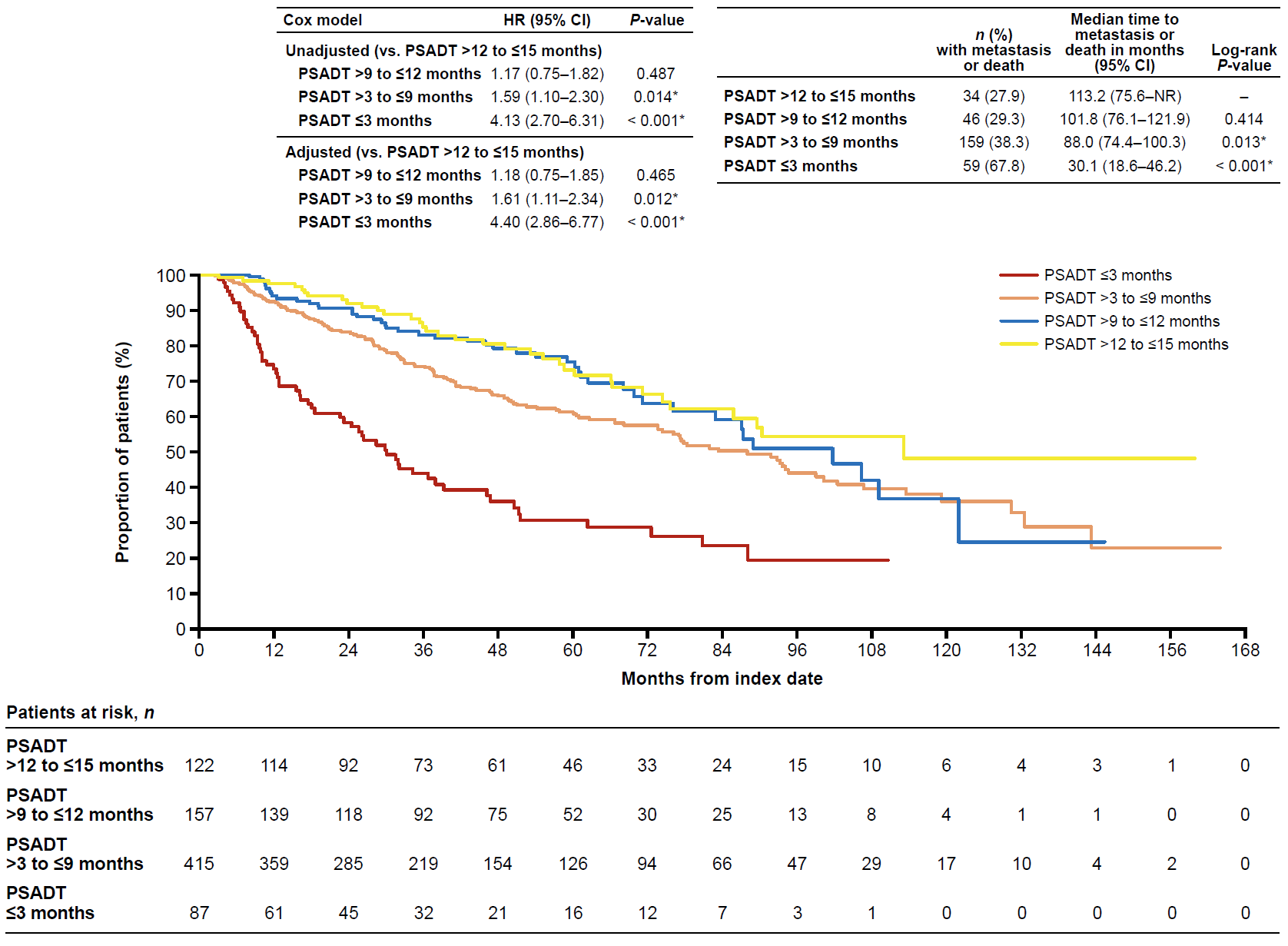
Supplementary Fig. S2. MFS after BCR among patients with nmCSPC by further PSADT cohorts.

The adjusted Cox proportional hazards models adjusted for patient demographics and clinical characteristics including age, age group (<60 [reference], 60–69, 70–79, and ≥80 years), race (White [reference], Black, Hispanic, and other), log(time from definitive therapy to index date), log(index PSA), modified Charlson Comorbidity Index, and index year (2006–2016 vs. 2017–2020).
**P*-value ≤ 0.05.
*BCR* biochemical recurrence; *CI* confidence interval; *HR* hazard ratio; *MFS* metastasis-free survival; *nmCSPC* non-metastatic castration-sensitive prostate cancer; *NR* not reached; *PSA* prostate-specific antigen; *PSADT* PSA doubling time.


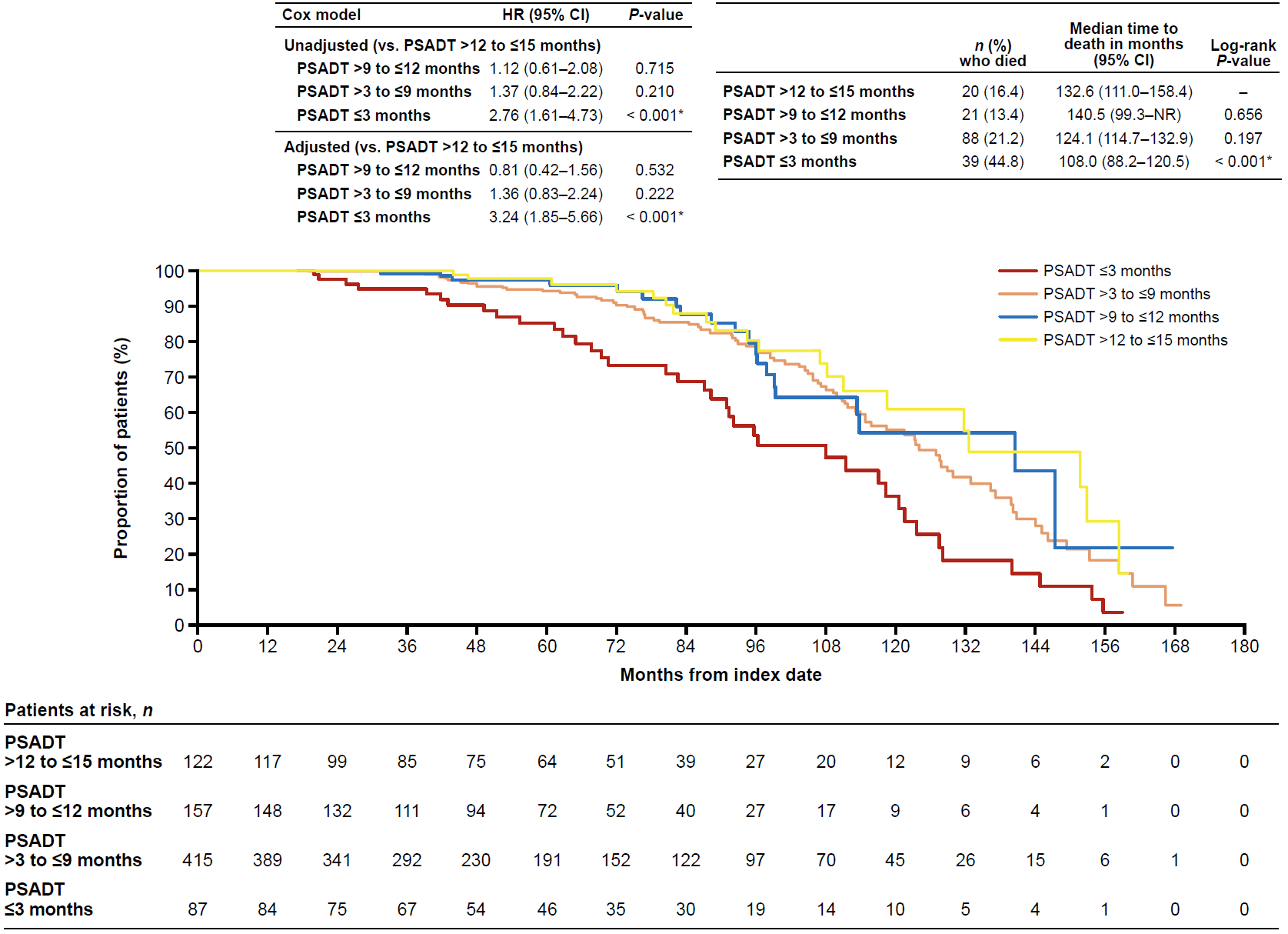
Supplementary Fig. S3. OS after BCR among patients with nmCSPC by further PSADT cohorts.

The adjusted Cox proportional hazards models adjusted for patient demographics and clinical characteristics including age, age group (<60 [reference], 60–69, 70–79, and ≥80 years), race (White [reference], Black, Hispanic, and other), log(time from definitive therapy to index date), log(index PSA), modified Charlson Comorbidity Index, and index year (2006–2016 vs. 2017–2020).
**P*-value ≤ 0.05.
*BCR* biochemical recurrence; *CI* confidence interval; *HR* hazard ratio; *nmCSPC* non-metastatic castration-sensitive prostate cancer; *NR* not reached; *OS* overall survival; *PSA* prostate-specific antigen; *PSADT* PSA doubling time.

Supplementary Fig. S4. Male-specific mortality by age (2019) – data from the National Center for Health Statistics, National Vital Statistics System, Mortality.


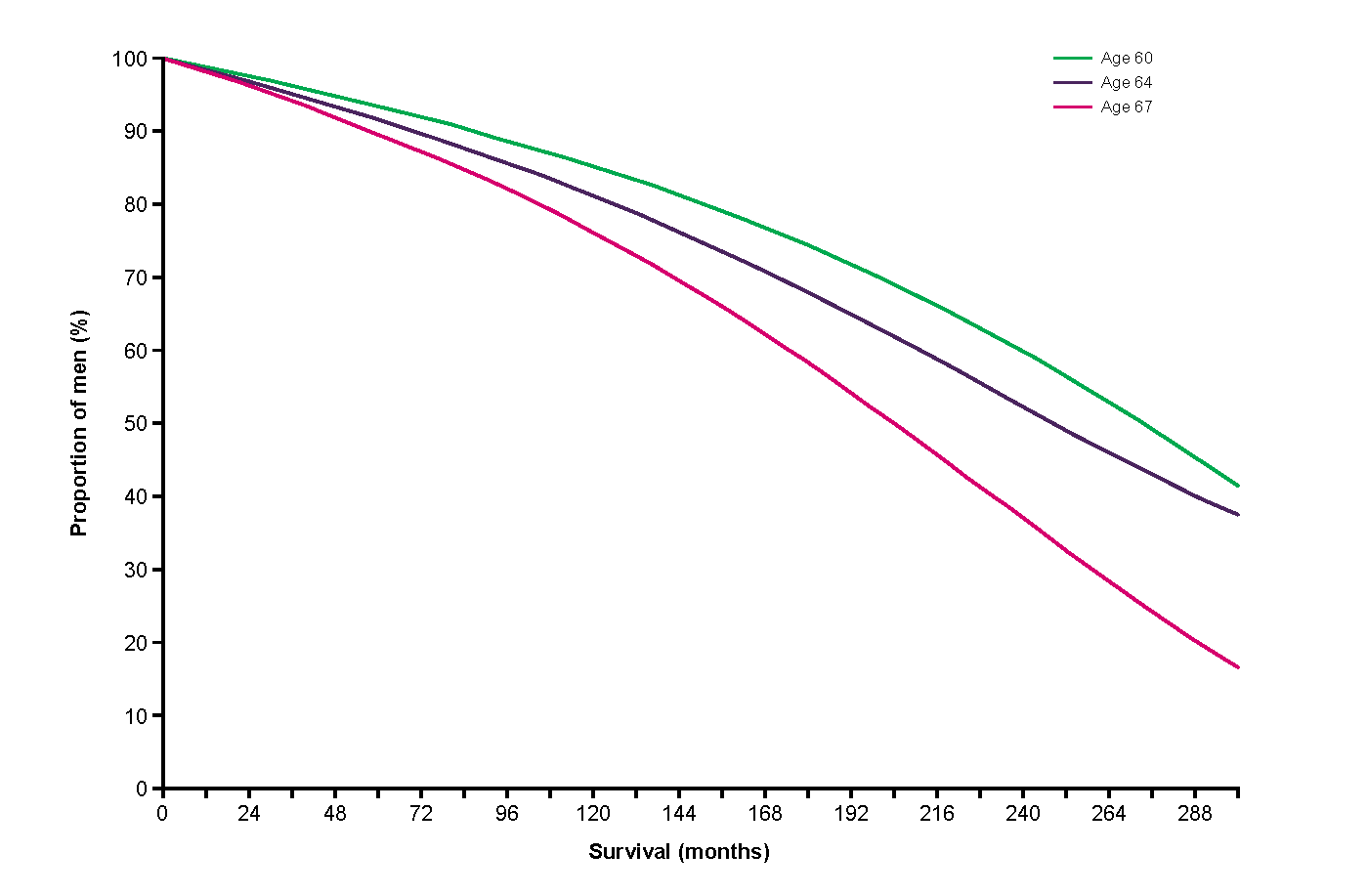

Supplement: Supplementary file 1 — Supplemental Material [file 41391_2024_894_MOESM1_ESM.docx]
